# The Embodied Crossmodal Self Forms Language and Interaction: A Computational Cognitive Review

**DOI:** 10.3389/fpsyg.2021.716671

**Published:** 2021-08-16

**Authors:** Frank Röder, Ozan Özdemir, Phuong D. H. Nguyen, Stefan Wermter, Manfred Eppe

**Affiliations:** Knowledge Technology, Department of Informatics, University of Hamburg, Hamburg, Germany

**Keywords:** embodiment cognition, grounding language, dialog, minimal self, reinforcement learning, developmental psychology, developmental robotics

## Abstract

Human language is inherently embodied and grounded in sensorimotor representations of the self and the world around it. This suggests that the body schema and ideomotor action-effect associations play an important role in language understanding, language generation, and verbal/physical interaction with others. There are computational models that focus purely on non-verbal interaction between humans and robots, and there are computational models for dialog systems that focus only on verbal interaction. However, there is a lack of research that integrates these approaches. We hypothesize that the development of computational models of the self is very appropriate for considering joint verbal and physical interaction. Therefore, they provide the substantial potential to foster the psychological and cognitive understanding of language grounding, and they have significant potential to improve human-robot interaction methods and applications. This review is a first step toward developing models of the self that integrate verbal and non-verbal communication. To this end, we first analyze the relevant findings and mechanisms for language grounding in the psychological and cognitive literature on ideomotor theory. Second, we identify the existing computational methods that implement physical decision-making and verbal interaction. As a result, we outline how the current computational methods can be used to create advanced computational interaction models that integrate language grounding with body schemas and self-representations.

## 1. Introduction

**Figure 1:**
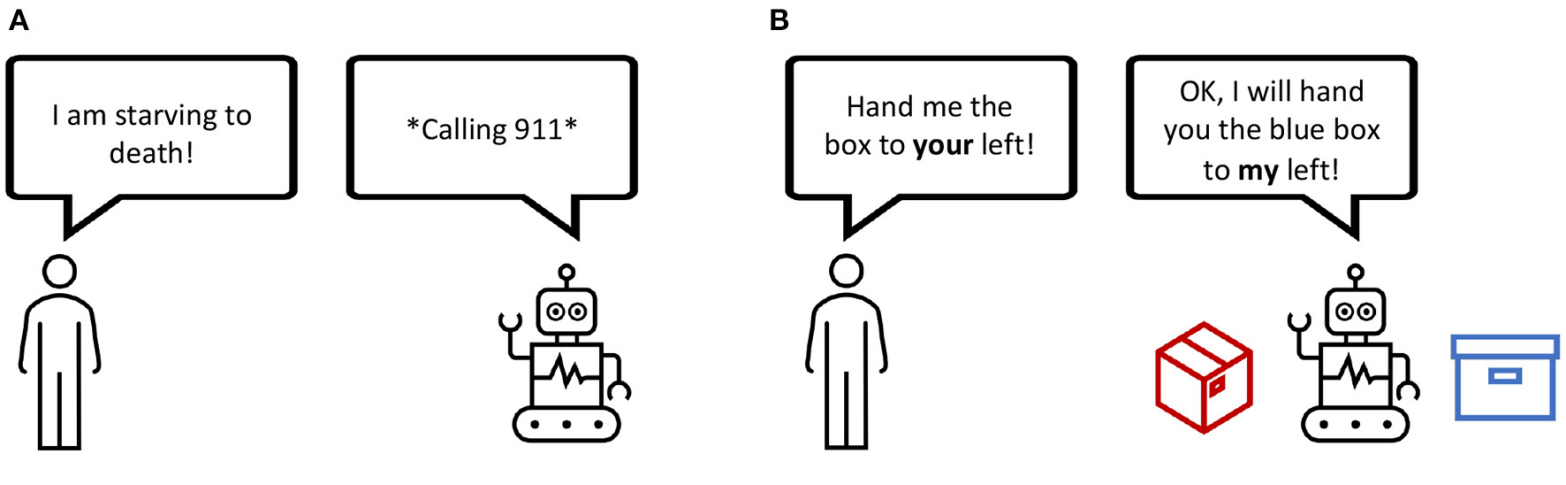
**(A)** Misunderstanding a metaphor, potentially due to the lack of a self-representation. **(B)** A robot using the self as point of reference to understand an instruction.

The human species has a unique communication system that involves verbal (e.g., speech) and non-verbal (e.g., gestures, facial expressions, body language) interaction with others. Despite cultural and social differences, participants in a conversation need to share a common conceptual view of the world and their embodied self. This is essential to have a common understanding, avoid misunderstandings, interpret metaphors (Feldman and Narayanan, 2004) (see Figure 1A), and for self-other distinction (Schillaci et al., 2013). A common conceptual view of the world is a consequence of the shared commonalities in how conversation partners ground language in their embodied interaction with the world (Barsalou, 2008; Madden et al., 2010). For example, the common conceptual view implies a self-representation that enables humans to solve tasks involving intrinsic spatial reference frames, like the one in Figure 1B. But how can humans learn appropriate representations of their body and, consequently, their self? Is the self a unifying principle that combines all the needed ingredients to solve both mentioned examples?

In this review, we will address these questions from an interdisciplinary perspective. Therefore, we will first discuss the cognitive and psychological background for self-representation and embodied language learning. Second, we will align this background with contemporary research in reinforcement learning. Herein, we focus on the cognitive mechanistic aspects of representation learning and behavior. We also appreciate insights from neuroscientific literature (Rizzolatti and Arbib, 1998; Kaplan, 2007; Madden et al., 2010), but we draw only occasional links to maintain a feasible scope for this article, we draw only occasional links to particularly relevant neuroscience background.

### 1.1. Embodied Language Learning

Human-robot interaction (HRI) is an active field of research where communication via natural language is an essential but also a very challenging component. In the past years, methods utilized machine learning to improve natural language processing (NLP), enabling decent interactions with virtual agents like Siri, Alexa, Cortana, and Google. These improvements are mainly due to utilizing large neural network-based language models (Vaswani et al., 2017; Devlin et al., 2019). However, these systems are limited to disembodied language processing, and therefore, cannot understand how natural language is situated in the physical world. For example, properties such as “heavy” or “hot” cannot be experienced without sensors, and they are important for robots interacting with humans. A robot should understand that hot things can hurt living beings and that not every person can lift heavy objects. There exists research on how robots can technically acquire and understand language through sensorimotor grounding (Steels et al., 2012; Spranger et al., 2014). However, in practice, this is still challenging for current computational models on robots as sensory inputs are imperfect, and natural language is full of ambiguities (see Figure 1A). For example, Steels and Loetzsch (2012) present research on how robots can establish new names for objects they see in an environment. They play a grounded naming game with a hardcoded cognitive system and vision, speech recognition, and pointing mechanisms. This is consistent with the concept of decoupling skill learning and language language grounding (Akakzia et al., 2021; Lynch and Sermanet, 2021) that we consider in this article.

**Figure 2:**
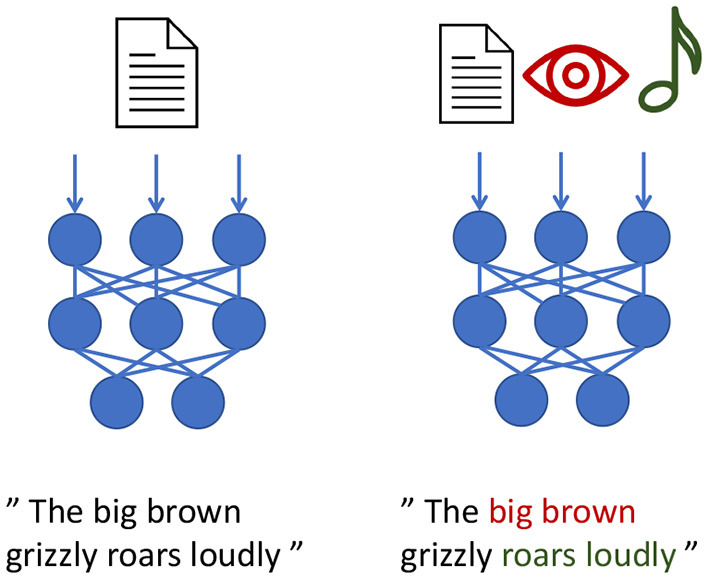
We illustrate an example using a neural text-processing model that integrates text only **(left)** and text in combination with vision and sound **(right)**. Possible associations or groundings are highlighted.

To address the problem of imperfect sensors and noisy perception, researchers and engineers often use crossmodal inputs following the notion of the duck test for deductive reasoning: “*If it looks like a duck, swims like a duck, and quacks like a duck, then it probably is a duck.”* (Hill et al., 2020; McClelland et al., 2020). Language models, even if showcased as extremely powerful like GPT-3 (Brown et al., 2020), are limited as they cannot make sense of *swimming* or what a *quaking* duck would sound or even look like. To fully understand what swimming and quacking are, an agent requires embodied and situated experiences to ground these concepts. This includes physical interaction with water and, preferably, cross-modal visual and acoustic sensory input to perceive the quacking. In other words, many of the existing language models like GPT-3 perform Natural Language *Processing* (NLP), but they lack the embodied grounding processes required for Natural Language *Understanding* (NLU). As a consequence, to understand language in the context of a dialog and to be able to interact physically with the world via actuators, it is critical to receive embodied multisensory inputs, such as vision, sound, and touch. Figure 2 illustrates a possible association between the language modality and other modalities (right side) compared to a model that cannot use such grounded connections. Understanding grounded language is critical for acting robots (Tellex et al., 2020) to perform dialog (Bordes et al., 2017) and HRI in general.

Many human skills can be acquired by explanation through language only. However, learning physical skills like a backflip is hard and costly to learn by verbal explanations only because it also benefits from the athletic experience. For example, Christiano et al. (2017) were able to teach an agent to do a backflip via simple feedback akin to basic language only, describing how good the agent is currently performing or what to improve. The key point is that learning skills through language require embodied concepts that recall motions and postures in context. For example, “*While jumping as high as you can, pull your legs towardz your body and throw yourself to the back; after a full rotation, land on your feet”* presupposes that the skill “*jumping”* is already known. Without such concepts, explaining the execution of a backflip, similarly to the example of Christiano et al. (2017), requires a vast amount of feedback or very detailed guidance to compensate for the lack of knowledge.

In summary, humans leverage embodied concepts built up during their lifetime, with language understanding always tightly connected to knowledge and experiences of the motor system (Fischer and Zwaan, 2008). Specifically, verbal descriptions like “*throwing a ball”* or “*jumping in the air”* excite the relevant parts of the motor cortex that are active for both hearing and executing. Therefore, language acquisition is strongly influenced by embodied experiences and the current context (McClelland et al., 2020).

### 1.2. Reinforcement Learning and Computational Language Understanding Methods

Reinforcement learning (RL) (Sutton and Barto, 2018) is a cognitively plausible and valuable framework to emulate infant-like learning, exploring the world with a trial-and-error approach based on rewards. RL-based agents are sometimes intrinsically motivated (Forestier et al., 2017; Colas et al., 2020; Akakzia et al., 2021; Hill et al., 2021). They imitate behaviors (Chevalier-Boisvert et al., 2019; Lynch and Sermanet, 2021), use hierarchical abstractions to decompose a complex task into simpler tasks (Oh et al., 2017; Eppe et al., 2019), and some of them can be trained with language to follow instructions (Hermann et al., 2017; Oh et al., 2017; Chaplot et al., 2018; Narasimhan et al., 2018; Chevalier-Boisvert et al., 2019; Hill et al., 2019, 2020, 2021; Jiang et al., 2019; Colas et al., 2020).

Reinforcement learning is also a promising method to implement dialog systems (Shi and Yu, 2018; Saleh et al., 2020) and language-driven interactive RL (Cruz et al., 2015; Chevalier-Boisvert et al., 2019). Commonly, language in RL (Luketina et al., 2019) is either used to provide an instruction (what to do) or to assist the learning of the agent with hints and descriptions (Narasimhan et al., 2018). Other methods describe the agent's environment purely in textual form, e.g., the agent's state in a dialog or text-based game (Côté et al., 2019; Madureira and Schlangen, 2020), which is a common setup for most conversational settings. For example, the simulator ALFWorld (Shridhar et al., 2021) was published with the goal to provide a learning environment where they combine the text-based knowledge obtained in TextWorld (Côté et al., 2019) is combined with visual inputs from ALFRED (Shridhar et al., 2020). Saleh et al. (2020) use hierarchical reinforcement learning (HRL) (Barto and Mahadevan, 2003) in an open-domain dialog, providing results that are comparable with the current state-of-the-art language models (Vaswani et al., 2017). As another example for language-driven RL, consider the research by Jiang et al. (2019), who use simplified language to communicate between a lower and higher layer of a hierarchical RL agent following language instructions.

The recent review by Uc-Cetina et al. (2021) illustrates the applicability of RL in NLP to some extent, such as machine translation, language understanding, and text generation. The authors also suggest considering embodiment (Heinrich et al., 2020), textual domain knowledge, and conversational settings. Bisk et al. (2020) focus further on embodiment and highlight the importance of physical and social context, more precisely, multimodal sensory experiences, to apprehend the coherency of words and actions. In an embodied dialog, the notion of technically combining the world state, i.e., the sensory inputs, with a linguistic state of a dialog, e.g., the context of the last *n* utterances, is crucial. We also see advances in multimodal reinforcement learning (Schillaci et al., 2013; Chaplot et al., 2018; Hill et al., 2019, 2020, 2021), integrating multisensory experience for explainability and improved training performance.

### 1.3. Scientific Rationale and Contribution of This Review

The work of Eppe et al. (2020) provides a thorough review of the hierarchical concepts for embodied problem-solving, but the authors do not consider language. Another related review about computational models of the self and body schemas has recently been presented by Nguyen et al. (2021). However, the authors do not consider language either. We address this gap by examining the challenges of embodied dialogs (Hahn et al., 2020) in the context of the self, combining the presence of language with other input modalities to learn appropriate hierarchical representations.

For our review, we hypothesize that a disembodied combination of the latest insights in multimodal data processing and language processing is not sufficient to enable full language understanding in dialogs between humans and embodied computational agents like robots. Instead, we hypothesize that an increased focus on the embodied self is important to enable computational agents with true language *understanding* capabilities beyond the mere computational *processing* of language. We investigate this hypothesis by addressing the following research questions:

*What are the cognitive components of the self, and why are they important for communication and dialog? Which components have been realized computationally, and how? Which are still missing?*

To address these questions, and as our main contribution, we look into recent articles defining the prerequisites of an artificial self (Schillaci et al., 2016; Georgie et al., 2019; Hafner et al., 2020; Nguyen et al., 2021) and relate these prerequisites with verbal and non-verbal dialog methods for computational agents and reinforcement learning. In 2, we survey the developmental processes of humans to ground language in embodied sensorimotor representations of the self and its surrounding world. In 3, we summarize existing computational methods that use grounded language to train an agent. In 4, we address our main hypothesis by summarizing and detailing why the self contains all the components that make robots better language learners and dialog partners. In addition, we provide a blueprint for combining the different existing computational techniques. These results are followed by a brief conclusion in 5.

## 2. Cognitive and Psychological Perspectives of the Communicating Self

The development of the human ability to perform bi-directional language-based dialog is a process over three interleaved stages. The first stage is sensorimotor development, where infants learn to align their perception with their motor skills (Paul et al., 2018) to acquire an understanding of the physical dynamics of their environment. Based on such low-level sensorimotor knowledge acquisition, humans develop embodied mental concepts in a second developmental stage to model their environment in higher-level preverbal conceptual representations (Feldman, 2006; Barsalou, 2008; Frankland and Greene, 2020). Such higher-level concepts are the foundation of language, which emerges with social interaction and communication during the third stage of development (Feldman, 2006; Kiefer and Pulvermüller, 2012). These three stages are not temporally distinct, but they co-develop. For example, verbal interaction demands additional low-level motor skills to produce phonemes using tongue, lips, and diaphragm. And social interaction leads to learning new conceptual representations that describe social interaction, e.g., in meta-communication. In the following, we will summarize the psychological and cognitive foundations of each of these stages.

### 2.1. Learning Sensorimotor Representations

From the very first month of birth, infants start developing a sense of their own body and its relation to other physical entities, such as objects and other living beings (Nguyen et al., 2021). The representation of their body in space that encodes positional and relational information is called the body schema (Holmes and Spence, 2004; Hoffmann et al., 2010). The body schema, or sense of body, is mainly shaped by proprioception, but visual information and other modalities (Wermter et al., 2009), including sound, vision, pain, and smell, also play a role (Anderson, 1972). The multimodality of the formation of low-level sensorimotor representations is very efficient for humans suffering from a lack of one or more senses. For example, visually impaired humans can build a rich conceptual understanding of words, objects, and the world, even without the visual sense (Nguyen et al., 2021). Generally, the absence of one or more modalities can be compensated by the other modalities, such as touch and sound. Therefore, multisensory integration is crucial for embodied cognition and learning concepts to represent the world.

Ideomotor theory postulates that the physical knowledge about multimodal sensorimotor contingencies is encoded as bi-directional action-effect associations (Shin et al., 2010). This implies that neural structures learn a mapping between actions and effects that enable humans to predict the outcome of actions and external events. The same structures enable humans to select an action based on a *desired* effect, i.e., a goal. The acquisition of ideomotor associations is enabled by observing and interacting with the world, learning principles such as occlusion, solidness, collision, gravity, and other physical events (Baillargeon, 2001).

Developmental psychology suggests that the acquisition of sensorimotor knowledge is guided by several forms of intrinsic motivation, including self-guided play (Sutton-Smith, 2001), curiosity (Oudeyer et al., 2007), repetition, and imitation (Wood et al., 1976; Paulus, 2014). Self-guided play implies that infants conduct their own experiments, e.g., dropping toys to discover forces like gravity, to extend their knowledge about the world and their own capabilities (Sutton-Smith, 2001). This behavior is closely tied to curiosity and active learning: infants often strive to encounter surprising and unpredictable situations to maximize their knowledge about the world (Schwartenbeck et al., 2019). More specifically, Schwartenbeck et al. (2019) state that active learning builds on minimizing the *unexpected uncertainty*, which can be described as the uncertainty about uncertainty. The authors exemplify active learning with a two-armed bandit problem where the reward of using one arm is low, but the agent knows that the probability for the low reward is high. The other arm has a low but unknown probability for a high reward. In this case, an agent will first try to resolve the unexpected uncertainty about the unknown probability for a high reward of the second arm by trying it. In general, it will collect samples of state transitions with a high unexpected uncertainty until it has a good estimate of the uncertainty.

This explorative behavior, however, must be balanced with striving for predictable action-state transitions, as described by the *free energy principle* (Friston, 2009). This principle implies that humans and other acting systems perform an *active inference* behavior and seek to encounter predictable situations. It describes long-term surprise as an upper limit for free energy and states that biological agents strive to minimize the free energy. At first glance, active inference seems to contradict the active learning behavior where agents strive to encounter uncertain and unpredictable situations to maximize their knowledge gain. However, since active learning seeks to encounter situations with a high *unexpected uncertainty*, i.e., uncertainty about uncertainty, this is in fact very compatible with active inference, which seeks to avoid situations with a high *expected uncertainty*. In other words, active learning is preliminary to active inference because it is required to learn a model about expected uncertainty.

Another form of intrinsic motivation is repetition: Biological agents exhibit behaviors that are not only goal-driven but exclusively conducted for the purpose of repetition to discover multiple possible ways of achieving a goal (Burghardt, 2006). For example, one can think about a child stacking blocks just for the sake of stacking rather than the goal of building a big tower. In the goal-driven case, repetition allows experiencing many ways of achieving the same desired outcome.^1^ Acevedo-Valle et al. (2020) point out that intrinsically motivated sensorimotor exploration is also related to imitation. The authors' proposed architecture highlights imitation-based learning of an infant in the pre-linguistic phase, being supervised by an instructor. They consider the simulation of a vocal tract as a comparison to what young infants do to produce vocal sounds when acquiring speech. Most robots do not have a vocal tract, but there exists research on modeling goal-directed behavior where the goal is to produce a certain vowel or syllable (Philippsen, 2021). Here, the authors consider the case of speech acquisition, where goal-directed explorative behavior uses sounds to learn vowels and syllables via *goal babbling* (Philippsen, 2021).

In summary, explorative play and active learning are the main drivers for learning to “know the unknown” (Vygotsky, 1967; Belsky and Most, 1981) and, more specifically, about the effects and uncertainties of actions (Nguyen et al., 2021). However, explorative behavior is balanced with the free energy principle, causing agents to strive for predictable situations. Other drivers of sensorimotor learning are imitation and repetition. Once enough knowledge is acquired, humans and other animals can use their rich conceptual knowledge for one-shot problem-solving (Eppe et al., 2020).

### 2.2. Formation and Grounding of Preverbal and Abstract Conceptual Representations

Language allows humans to express thought. However, explicit verbal language is not a prerequisite for thought—there exists a preverbal hierarchical system of abstract mental concepts to enable thought (Frankland and Greene, 2020).

#### 2.2.1. Representational Abstraction

The human mind constantly performs inference on multiple layers of representational abstraction (Clark, 2016). The theory of embodied cognition suggests that the higher levels of abstraction emerge from the sensorimotor interaction of the lower levels (Barsalou, 2008; Lakoff and Johnson, 2010; Tani, 2016). Already during the first year of a human's life, sensorimotor abstraction leads to higher-level preverbal concepts that enable problem-solving and the understanding of simple language (Mandler, 2004). These concepts are grounded in sensorimotor experiences and perception, being later on shaped by our acquired language. Cognitive sciences often refer to such preverbal general concepts as *image schemas* (Lakoff and Johnson, 2010; Turner, 2015) or, in a more linguistic context, *semantic frames* (Barsalou, 2008; Gamerschlag et al., 2014).

How exactly such concepts are represented in biological neural structures remains largely unknown. In particular, there is a lack of research concerned with the semantic compositionality of mental concepts. There exists phenomenological research from the cognitive sciences community to model compositional high-level concept formation (Lakoff and Johnson, 2010; Turner, 2015; Eppe et al., 2018). On the other end of the spectrum, there also exists very low-level neuroscientific research showing the compositionality of distributed neural activation patterns via neuroimaging (Haynes et al., 2015). Between these extremes, there is some very interesting work related to binding neurons (Shastri, 1999) that can potentially model semantic role-filler bindings known from cognitive linguistics. The event segmentation theory (EST) is a biologically plausible model to explain action abstraction based on prediction errors (Zacks et al., 2007). However, to the best of our knowledge, no computationally verified and functional unifying theory integrates the cognitive sciences and linguistics perspective on symbolic compositional mental representations with the neuroscientific perspective of representing mental concepts as distributed neural activation patterns.

#### 2.2.2. Abstract Mental Concepts for Language and Creative Thought

Abstract preverbal concepts are not only critical for language acquisition, but they are also very important for creativity (Turner, 2015). For example, consider the metaphorical concepts of files and folders of a computer's operating system: the terminology for these concepts comes from the pre-digital age, originally from non-electronic paper-based files and folders. Blending this terminology with the tree-based algorithmic pointer concepts behind a computer's file system was a creative act that made it possible to align a human's pre-existing conceptual system with new technology and helped to improve the usability of early operating systems like Windows 95. Confalonieri et al. (2015, 2016, 2018) and Eppe et al. (2018) demonstrate the importance of such concept blending with a functional computational model that allows an artificial agent to combine two known concepts to new concepts with emergent useful and aesthetic properties. The authors show how the new blended concepts lead to the creative and serendipitous discovery of lemmas required for mathematical proofs and the automated (re-)discovery of famous chord progressions in jazz music.

### 2.3. Embodied Language Acquisition

Preverbal and abstract semantic concepts are the basis for language. Since abstract concepts emerge from low-level sensorimotor interaction, the body and environment have a great impact on our thinking and language acquisition (Feldman and Narayanan, 2004). Several studies highlight that hearing or reading language about action and perception activates related areas of the brain, showing that there are neural representations reflecting an individual's way of performing actions when heard (see the overview by Willems et al., 2010 or the work about the mirror system by Rizzolatti and Arbib, 1998). This is compatible with ideomotor theory (Shin et al., 2010) and mental simulation theory, which claims that humans simulate actions unconsciously within those areas of the brain responsible for motor planning. As a result, there exists an embodied mental semantics (Feldman and Narayanan, 2004; Steels, 2007; Willems et al., 2010), implying that living entities with different kinds of bodies simulate in different ways. For example, consider the difference between right- and left-handed people, using the contrary sides of the premotor cortex.

#### 2.3.1. Language Acquisition as Resolution of Mismatches

Mandler (2004) describes the preverbal phase in infants as dominated by general conceptual knowledge that is in a mismatch with the language we understand and start to use at the age of 9 months. General conceptual knowledge is required to execute goal-directed actions, understand spatial relationships and the difference between objects and animals. The conceptual knowledge is also important to derive non-trivial intentions of conversation partners (Trott et al., 2016). Consequently, when language becomes more important during a toddler's early life, there is a need to compensate for the mismatch between the rich self-acquired conceptual knowledge and the words used to describe the world. For example, toddlers would assign the word dog to a fox since they do not yet have the language to differentiate them more precisely (Mandler, 2004). Similar to machine learning models with the objective of classifying foxes, wolves, and specific breeds of dogs distinctively, a child would pay at some point closer attention to the details if the appearance is different, but the describing word stays the same (Mandler, 2004). One can also think about the attributes mentioned, like *black cat, red car*, or *big dog*, to accentuate a specific property, helping with the mapping of words to organize categories (Waxman and Markow, 1995). Mainly using a mixture of receptive language and producing words and simple sentences allows them to learn about things being said to and about them. Especially parents often explain to their children what they are doing, allowing them to learn word mappings to actions and objects nearly automatically, known as perceptual learning (Mandler, 2004). There is also a lot of imitation involved, e.g., replicating actions of social partners, repeating perceived utterances, or recalling sentences in a specific context.

There are still open questions at which point in time infants are capable of learning specific differences, especially those that are hard to grasp, like varieties between similar-looking plants that are not that frequently experienced in their daily life (Mandler, 2004).

#### 2.3.2. Toward Narrative, Egocentric, and Goal-Directed Language

When the first form of language is learned, infants tend to use egocentric speech, where they narrate their own activities (Piaget, 1926). Even though they do not have fully learned fluent language like adults, they use their present concepts and actively reinforce their speech in their own doing. This is different from babbling from an earlier stage, where the overall learning goal is to explore and correct their internal motor model of speech production with respect to adult language heard (see section 2.1). Furthermore, after infants learn a first basic corpus of language, they start using it to describe their intrinsically motivated goals. This can happen by just saying the word “arm” to tell their caregiver that they want to be picked up or by issuing more complex multi-word sentences of the form “I want X,” where the “I” reflects an emerging concept of the self (Georgie et al., 2019). Such goal-directed utterances to caregivers are among the first language-based communication situations.

#### 2.3.3. The Self and Communication

Language is very effective when it comes to communicating with other humans. The efficiency stems from the compositional structure of natural language. Most natural languages build on a finite vocabulary in the order of magnitude of 100,000 to 200,000 actively used words that can be composed to express an intractable number of different sentences and meanings. Our acquired knowledge about grammar, syntax, and semantics enables us to understand most of these compositions, even if we have never heard them before. For example, you may never have heard the sentence “She sneezed the napkin off the table.”, but your knowledge about English grammar enables you to correctly understand it. This demonstrates that language is an important cognitive tool to convey meaning (Mirolli and Parisi, 2011; Colas et al., 2020; Eppe and Oudeyer, 2021). However, the self described in recent literature (Schillaci et al., 2016; Hafner et al., 2020; Nguyen et al., 2021) is also important for embodied dialog. The self builds upon the actor's capabilities to sense its own body and the environment. It is, therefore, characterized by the response to actions and predictions of the internal model (Schillaci et al., 2016; Hafner et al., 2020). Grounded language in the context of the self refers to the context of these senses. For example, the phrase “*Hand me the box to your left.”* (see Figure 1B) requires the robot to classify and detect the desired object (Matuszek et al., 2012) that is next to itself. Once the sentence is understood, a sequence of motor controls needs to be executed to fulfill the instruction. While the language already contains important contextual information, such that it is a box and not another object, which requires different balancing and grasping, the clue “*next to you”* suggests the object be in reachable distance, also described as peripersonal space (Nguyen et al., 2021) with respect to the self. The executed actions are conditioned on the initial instruction of handing over the bottle. The theory about the mirror system by Rizzolatti and Arbib (1998) hightlights the linkage between language and action representations (Wermter et al., 2009): Humans can merely recognize the intent of others by observing their behavior, e.g., if someone is approaching another person offensively. Intention recognition, however, plays a core role in communication and dialogs. We build on this neuroscientific perspective to underpin our claim that a self- and other-manifold is essential for embodied dialogs.

Current computational methods cannot effectively learn a theory of mind with the concepts of *you* and *me*. Therefore, they fail to learn robust and general behaviors. We suppose that this gap is due to a lack of understanding of “the self” (Hafner et al., 2020), and how it is defined in the context of “the other.” Specifically, we suggest that a self-other projection model is critical for empathy and a theory of mind to map an observed other agent, along with its semantic properties and relations, to the self and its semantic properties and relations.

In the following section, we will address this gap by investigating the computational language acquisition models that exist and summarize how they relate to the cognitive, psychological, and neurological perspectives on the communicative self.

## 3. Computational Methods

Current advances in neural language modeling accelerated the research progress in many NLP tasks (Vaswani et al., 2017; Devlin et al., 2019). Successful pre-trained one-shot models like GPT-3 (Brown et al., 2020) have many useful applications. Remarkable results were presented with the recently introduced successor version of GPT-3, named DALL-E (Ramesh et al., 2021), which learns visual-linguistic representations that align textual with image inputs to generate, based on text descriptions, samples of new pictures, showing up compositional conceptualization. For example, the sentence “*a red table in shape of a pentagon”* lets the model generate samples of red pentagon-shaped tables based on its learned multimodal representations. However, models like GPT-3 and DALL-E consider only disembodied language learning without any sensorimotor grounding because, unlike robots, they cannot physically interact with the world. Insights for grounded language learning in robotics (Heinrich et al., 2020) with sequential decision-making settings (Akakzia et al., 2021; Lynch and Sermanet, 2021) and embodied cognition (Feldman and Narayanan, 2004; Fischer and Zwaan, 2008) accentuate the need for embodied grounding. This includes physical interaction and multiple sensory modalities to develop systems that understand language more like humans (Anderson, 1972; Wermter et al., 2009; McClelland et al., 2020). Additional prerequisites for modeling a communicative self requires curiosity, body representations, and predictive processes (Hafner et al., 2020; Eppe and Oudeyer, 2021). In reinforcement learning, there is a body of research (Pathak et al., 2017; Dean et al., 2020; Nguyen et al., 2020; Röder et al., 2020), containing these components. However, to the best of our knowledge, these prerequisites have not yet been combined with language and the self in mind. Overall, there is a lack of research methods that regard the self in the area of RL, explicitly making use of language in embodied dialogs (Hahn et al., 2020). This section reviews methods that partly satisfy the requirements but still miss at least one of the desired components. Furthermore, we provide an outlook on what needs to be recombined or is missing to learn self-other representations in embodied dialogs.

### 3.1. Formal Background

Reinforcement learning (Sutton and Barto, 2018) is based on a Markov decision process (MDP) defined by a tuple (S,A,T,R,γ), where S is the space of all possible states, A the space of all possible actions, T:S×A×S→[0,∞) the transition probability function, R:S×A→ℝ the reward function, and γ∈[0, 1) is the discount factor. The transition function represents a probability density of transitioning to a following state $s^{\prime }\in S$, when executing action a∈A, being in state s∈S. The reward function describes the immediate real-valued reward obtained when transitioning to the next state. The overall objective is to find a policy π that selects actions, π(*a*_*t*_|*s*_*t*_), to maximize the expected discounted reward $\overset{T}{\underset{t=1}{\sum }}{𝔼}_{\pi }\left[\gamma^{t}R\left(s_{t},a_{t}\right)\right]$ for every time step *t*.

#### 3.1.1. RL and Imitation Learning

The definition of the MDP, as mentioned earlier, also applies to the framework of imitation learning (IL) (Atkeson and Schaal, 1997; Lynch and Sermanet, 2021), where the learner only has access to a sequence of state-action pairs (*s*_1:*T*_, *a*_1:*T*_) of an expert—hence the optimal or suboptimal policy—without knowing the reward function *R*.

#### 3.1.2. Language as Goal

In this review, we consider papers that also augment this setup with a set of goals G and condition the action-selection of the policy based on the present state and goal, π(*a*_*t*_|*s*_*t*_, *g*_*t*_), also named as goal-conditioned RL (Oh et al., 2017; Chaplot et al., 2018; Chevalier-Boisvert et al., 2019; Jiang et al., 2019; Colas et al., 2020; Röder et al., 2020; Akakzia et al., 2021; Lynch and Sermanet, 2021). One way of integrating language into the augmented MDP, is to learn a mapping from language to goal, *m*(*l*_*t*_) → *g*_*t*_. Another approach is to provide extra input to the policy or concatenate and extend the dialog state as a combination of language and world state, $s_{t}=\left[s_{t}^{world},s_{t}^{dialog}\right]$. However, these are technical questions that we do not further consider within this article.

### 3.2. Recent Advances in Reinforcement Learning With Language

Modeling language occurrences in a simulated environment is not obvious to implement, and using human-annotated linguistic training data is usually inefficient and costly. It is also a very specific design decision, how complex the sentences and how limited the vocabulary of words used to train the agent are (see section 3.3).

The review of Luketina et al. (2019) provides an overview of the recent progress of language-processing RL agents where researchers explore possibilities of integrating neuro-plausible principles, such as intrinsic motivation (Forestier et al., 2017; Colas et al., 2020), to foster language learning. Many approaches benefit from mapping instructions to action sequences (Branavan et al., 2010; Misra et al., 2017), latent plans (Lynch and Sermanet, 2021), semantic goals (Akakzia et al., 2021), and internal abstractions (Jiang et al., 2019). In section 3.3, we further examine the possibilities of providing language data to artificial agents that learn from sparse rewards as successfully presented by recent approaches (Luketina et al., 2019; Dean et al., 2020; Akakzia et al., 2021; Lynch and Sermanet, 2021). We see a trend of detaching from the traditional MDP formulation and integration imitation-based (Lynch and Sermanet, 2021) and self-supervised methods (Akakzia et al., 2021) into a learning framework to autonomously acquire motor skills and language understanding with minimal human intervention. We draw inspiration from the intrinsically motivated learning of infants, like mentioned in 2, based on a cognitive and developmental perspective.

#### 3.2.1. Dataset-Driven RL Methods

Generally, methods make use of sparse goal annotations (Akakzia et al., 2021; Lynch and Sermanet, 2021) or generate scene-dependent descriptions (Narasimhan et al., 2018; Hill et al., 2021) and instructions (Hermann et al., 2017; Oh et al., 2017; Chaplot et al., 2018; Chevalier-Boisvert et al., 2019). Such methods often build on a previously collected fixed dataset. Therefore, most language-conditioned and language-assisted agents are limited in these settings as they do not reveal behavioral diversity, sticking to a poor set of discovered solutions. This is a problem for embodied agents in dialogs and HRI, with potential uncertainties and inaccuracies coming with dynamics of the physical world. Furthermore, many do not consider all the available modalities to build rich and robust representations, including self-representation (Nguyen et al., 2020). Recent work shows that RL with language needs another type of benchmarking, similar to supervised learning, evaluating the agent on unseen tasks, objects, and instructions (Hill et al., 2020). Otherwise, one could not prove the generalizability of learned feature representations that encode concepts and meanings that are relevant. Especially for our case, we consider an embodied conversational setup with an agent and a human communicating, where having a self-other representation is beneficial if not crucial (see Figure 1B).

#### 3.2.2. Adding Dynamic Data and Language Grounding

Using datasets only to train RL-based dialog agents creates limitations. However, datasets can be used for pre-training when a basic understanding of language is necessary to solve a certain task. They can also be augmented with other data, such as demonstrations and pre-trained word embeddings. This can also be combined with other learning methods, such as inverse RL.

Interesting perspectives in this direction are covered in the work of Luketina et al. (2019): The authors consider language-conditioned RL, where language processing is inevitable to fulfill a task because either the state space or action space contains language. A sequence of instructions needs to be followed, telling the agent what to do or which goal to accomplish. The authors argue that following high-level instructions has a strong connection to hierarchical RL (HRL) (Oh et al., 2017; Jiang et al., 2019), decomposing the overall dialog into a sequence of subtasks (Röder et al., 2020).

Another approach presented in the same study (Luketina et al., 2019) is to infer the reward function from the present instructions, especially where no external reward is available, but a set of demonstrations is present. A suitable strategy in such a case is inverse RL (Ng and Russell, 2000). An optimal or suboptimal policy trajectory is used to reconstruct the underlying reward function *R* as the origin of the demonstration policy's behavior. Unlike behavior cloning, as the simplest form of imitation learning, a goal-achievement reward function could be learned (Colas et al., 2020), which could also be helpful for intrinsically motivated- and transfer learning.

Next, Luketina et al. (2019) consider language-assisted RL, which is also partly related to language-conditioned RL, where language eases the learning and is not required to solve a task. Here, language is descriptive and contains assisting clues for the agent, e.g., “be careful with the delicate plates” (as additional hint before the agent tries to pick them up) or “to open a door, it needs to be unlocked with a key” (the agent is facing a door and is stuck or randomly tries to find a solution). This setting requires the agent to retrieve the relevant information for a given context, where a grounded language understanding is inevitable.

Lynch and Sermanet (2021) show that combining imitation learning with pre-trained word embeddings enables zero-shot learning. Approaching problems with pre-trained models like BERT from Devlin et al. (2019) can circumvent the effort to train so-called “*tabula rasa”* RL agents (Luketina et al., 2019), that is, agents that need to learn language and sensorimotor control simultaneously from scratch. Conclusively, language is a vehicle for transfer learning, as it encodes world knowledge distilled from large text corpora (Devlin et al., 2019; Brown et al., 2020). We believe that language in RL (Luketina et al., 2019) should focus on aligning its sensorimotor representations, learning from multisensory inputs (Hill et al., 2021; Ramesh et al., 2021) that exploit and ground the present compositional and hierarchical linguistic concepts.

### 3.3. Language Data for RL Agents

When infants interact with their caretakers and the world, they receive visual, auditory, and haptic feedback. In addition, they are also exposed to linguistic utterances and speech in the context of this interaction. In machine learning, this corresponds to interactive RL (Cruz et al., 2015). However, as opposed to human infants that can learn from a few examples very efficiently, RL agents require large amounts of interaction data to learn a reasonable behavior. Furthermore, the required presence of a human partner in the training process is still costly and time-consuming. For this review, we consider approaches (1) that can efficiently collect language before training (Chaplot et al., 2018; Narasimhan et al., 2018), (2) that can automatically generate linguistic instructions at training and testing time (Hermann et al., 2017; Chevalier-Boisvert et al., 2019; Jiang et al., 2019; Hill et al., 2020, 2021), and (3) that require only minimal linguistic input for an agent in the learning process (Colas et al., 2020; Akakzia et al., 2021; Lynch and Sermanet, 2021).

#### 3.3.1. Gathering Data in Advance

Approaches that fall into the first category, such as Narasimhan et al. (2018) and Chaplot et al. (2018), gather language data in advance. Narasimhan et al. (2018) utilize Amazon Mechanical Turk (Buhrmester et al., 2011) to collect descriptions of entities (their roles or behaviors) in different game environments—Amazon Mechanical Turk offers a crowdsourcing website where researchers can hire so-called crowd workers to collect large amounts of data easily and rapidly for a particular task. For each game environment, annotators are shown videos of gameplay and asked to describe entities in terms of their role or behavior, whereby a set of descriptions are collected. It is important to note that the annotators are prompted to give descriptive information about the entities rather than instructive information, which may help the agent complete the given task. The agent, in turn, exploits the appropriate set of descriptions in an end-to-end learning process to reach its goal for a given environment. Chaplot et al. (2018), on the other hand, manually create 70 instructions that prompt the agent to navigate in a 3D game environment and find the target object. Each instruction follows the template “Go to the X” where X is an object with its properties such as “green torch,” “tall blue object” etc.

#### 3.3.2. Automated Generation of Verbal Instructions

The second category approaches, such as Chevalier-Boisvert et al. (2019) and Jiang et al. (2019), can automatically generate language input during training and testing. Jiang et al. (2019) use the *CLEVR* language engine (Johnson et al., 2017), which programmatically generates scenes of objects and language descriptions/instructions. This also requires the agent to learn a language-conditioned policy in an end-to-end fashion (see section 3.2). In this sparse-reward setting, the authors use *hindsight instruction relabeling* (Jiang et al., 2019) to improve sample efficiency. Chevalier-Boisvert et al. (2019) introduce a synthetic language, the Baby Language, which has a systematic definition with combinatorial properties. Albeit a proper subset of English, the Baby Language has 2.48 × 10^19^ possible instructions. It has a special grammar based on which synthetic instructions with different actions (pick up, drop, move), colors, objects, and locations (e.g., “move the green ball next to the blue box”) can be generated.

#### 3.3.3. Training With Sparse Data

Lynch and Sermanet (2021) and Akakzia et al. (2021) are considered in the third category because they require only very little language data for the agent during the learning process. Lynch and Sermanet (2021) introduce multicontext imitation, which allows flexibility to use paired state-action language data for less than 1% of the examples to train the agent. They pair play data with human language, which they call *hindsight instruction pairing*. They randomly select a robot behavior from play and ask human annotators to describe it with the most suitable instruction, with the question “Which language instruction makes the trajectory optimal?” in their mind. From goal image examples, a paired goal image and language dataset is created that consists of short trajectories paired with unrestricted instructions collected from human annotators. Akakzia et al. (2021) utilize a synthetic social partner that describes the actions of the robotic arm manipulating objects in a simulator.

The first two category methods that we review in this paper do not strictly follow the approach we propose in this work. Many of them integrate the language data directly into the simulation. For our approach, we consider two phases (see **Figure 4**) where data collection is important: *skill learning* and *language grounding*. As a first phase in the *skill learning* (Akakzia et al., 2021), the agent curiously collects data to learn goal-directed behaviors, similar to infants in their preverbal phase (see 2), shaping their body schema (Nguyen et al., 2020). Subsequently, a social partner or caregiver provides the language to be grounded in the present goal-directed motor skills. Like infants, the agent should align and learn word meanings with the corresponding action effects. We consider a sparse annotation like applied in Lynch and Sermanet (2021) with *hindsight instructions* of < 1% of demonstrations—proposing the optimal instruction after the fact—or behavior annotations like (Akakzia et al., 2021) with only 10% of episodes as plausible approaches in line with the sparse utterances an infant experiences.

### 3.4. Decoupling Language Grounding From Skill Learning

**Figure 3:**
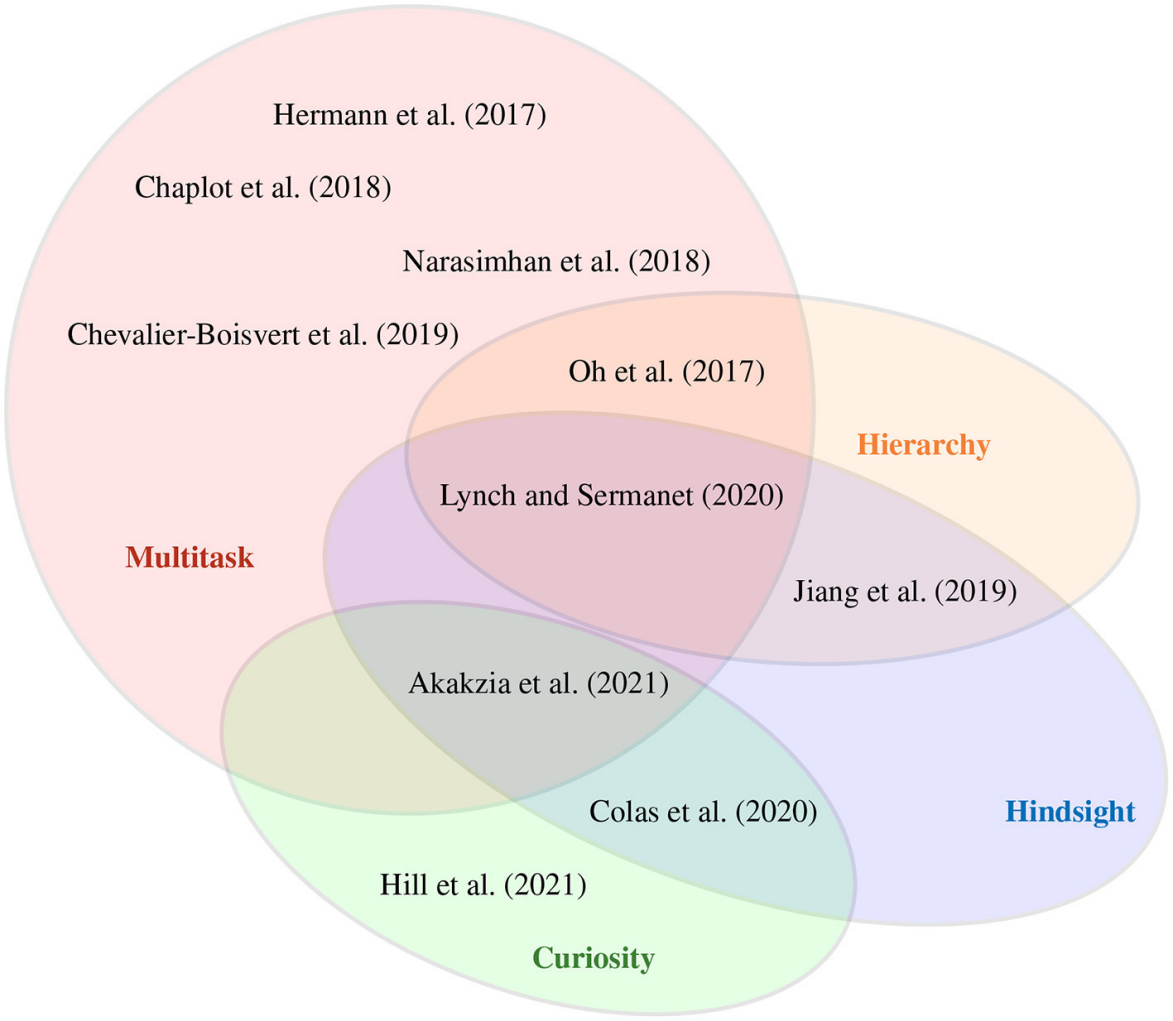
A selection of reinforcement learning methods which we categorize according to their properties. *Multitask* RL involves methods that learn a policy to solve and transfer knowledge between different tasks. *Hindsight* learning allows to create and learn from imagined—(Colas et al., 2020) and relabeled goals (Akakzia et al., 2021). Methods using a *hierarchy* of policies/models are employed for temporal abstractions (Jiang et al., 2019; Lynch and Sermanet, 2021). *Curiosity* serves as an intrinsic signal to utilize self-supervision and overcome sparse extrinsic feedback (Colas et al., 2020; Akakzia et al., 2021; Hill et al., 2021). The methods with the largest overlaps, namely Lynch and Sermanet (2021) and Akakzia et al. (2021), integrate both essential and cognitive plausible mechanism.

**Figure 4:**
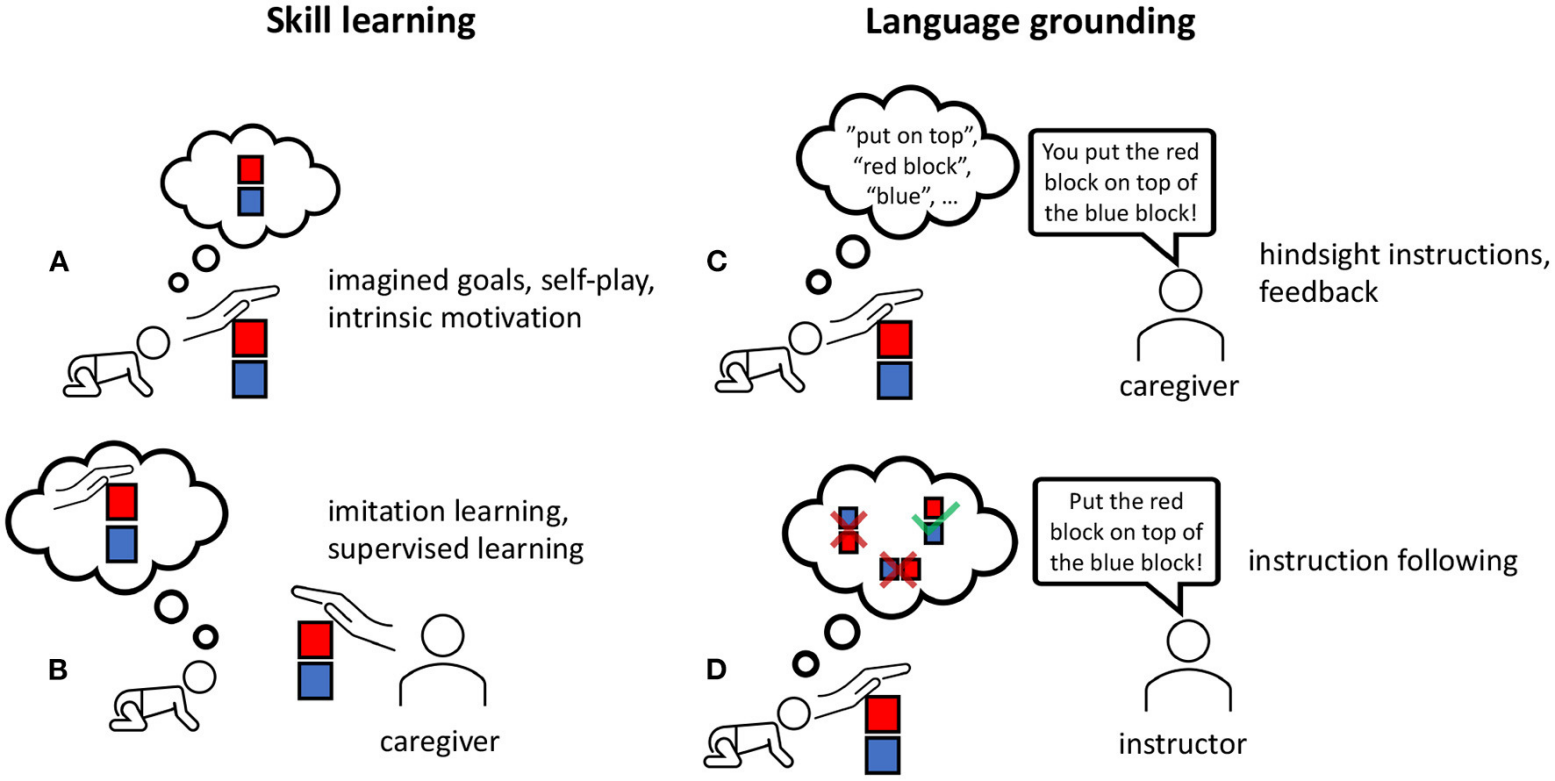
We accentuate the learning phases of current methods (Akakzia et al., 2021; Lynch and Sermanet, 2021) that have a grounded language acquisition by first learning behaviors/skills **(A,B)**—be it via imitation learning or intrinsic motivation—and following this, ground language in actions by receiving instructions or feedback from a caregiver **(C,D)**.

We visually summarize our review of research with respect to different approaches used in language-driven RL in Figure 3. The figure illustrates the underlying techniques, showing the most overlaps with respect to the categories *multitask, hierarchy, curiosity*, and *hindsight* in RL. Based on this categorization, we identify two methods that we consider most appropriate to address the research question of this article, namely Lynch and Sermanet (2021) and Akakzia et al. (2021). Among the approaches we discuss here, only these two consider the decoupling of learning skills and grounding language for an embodied robot in a 3D environment. This is important because in order to benefit from insights of preverbal goal-conditioned behavior in human infants (Wood et al., 1976; Mandler, 2004), artificial agents should be able to learn sensorimotor skills without the presence of language right at the beginning of the learning process. For our following discussion, we perform an in-depth analysis of these two methods. Based on the insights from 2, we split the overall learning into two phases, as shown in Figure 4: *skill learning* and *language grounding*.

#### 3.4.1. Skill Learning

The skill learning phase (Figures 4A,B) treats the sensorimotor skill learning as (a) learning those skills independently via imagined goals or concepts like self-play and intrinsic motivation or (b) emulating the behaviors of a caregiver via imitation or supervised learning. In the first case (Figure 4A), the agent could learn via intrinsically motivated play or mental problem-solving (imagination) to explore possible block configurations (Akakzia et al., 2021). This is similar to how an infant learns by exploring the environment while interacting with the objects around.

In the second case (Figure 4B), the agent could learn by imitating the caregiver (Lynch and Sermanet, 2021). Lynch and Sermanet (2021) conducted imitation learning on a dataset of play data. One benefit of play data is the unrestricted setup without solving any particular tasks. In their setup (Lynch and Sermanet, 2021) have a fixed robot arm in front of a desk with buttons, a cupboard, and other objects. The dataset is collected by recording the proprioceptive inputs, images from the camera, and executed motor control. Herein, the agent benefits from a knowledgeable human collecting the data. This yields a dataset of diverse and curious behaviors, including knowledge about object affordances.

#### 3.4.2. Language Grounding

In the second phase (Figures 4C,D), learning a grounded language is achieved by providing feedback or instructions. In Akakzia et al. (2021), a social partner—in our case, a caregiver (Figure 4C)—provides linguistic feedback, describing the behavior of the agent in hindsight. The social partner provides a description that considers a change in spatial relations between any two objects from the starting configuration to the final in the scene. Language grounding is achieved via a language-conditioned goal generator (LGG) which is implemented as a conditional variational autoencoder (Sohn et al., 2015): given an initial configuration and a description, LGG generates a corresponding final configuration, the goal for the agent to achieve. Resampling from the LGG allows the agent to solve the instruction in different ways, resulting in a diverse behavior (see section 2.1). Similar to Lynch and Sermanet (2021), only a small fraction of the author's dataset is annotated with instructions. These are provided in hindsight: after observing a particular behavior of the agent, the human provides the optimal “*hindsight instruction”* that would evoke this behavior.

Lynch and Sermanet (2021) extend the learning from play (LfP) approach (Lynch et al., 2020) by pairing experienced trajectories with natural language instructions, which they coin as LangLfP. They introduce *multicontext imitation* to train a single policy on both image and language goals. Multicontext imitation refers to training a single policy on shared latent representations of goal image and natural language datasets using image and language encoders. Multicontext imitation endows the approach with the flexibility to use paired state-action language data for less than 1% of the examples to train an agent. Having the ability to learn from sparsely annotated data corresponds with how infants learn in the real world with very little feedback from their caregivers. The trained agent can relate language to low-level perception, perform visual reasoning and solve a complex sequential decision problem. As a result, it can follow non-expert human instructions to perform object manipulation tasks in a row.

Lynch and Sermanet (2021) also exploit a large-scale pre-trained language model (Vaswani et al., 2017; Yang et al., 2020) to encode linguistic input; before feeding the language input to the network, they transfer it to a semantic vector space by using the pre-trained language model as an encoder. In this manner, the approach can handle unseen linguistic inputs such as synonyms, as well as instructions in 16 different languages. We suppose that training instruction-following and training dialog are suitable tasks for fine-tuning a pre-trained agent (Figure 4D). Moreover, continuing to learn a pre-trained mapping of new objects to concepts appears to be a promising future approach to consider (Hill et al., 2021).

## 4. The Self in an Embodied Dialog

In this section, we propose the computational components of an embodied dialog agent, informed by the above analysis of skill learning and language grounding and inspired by the recent work about self-representations of Hafner et al. (2020) and Nguyen et al. (2021).

Naively, testing the capabilities of a language-aware agent could already involve tasks and instructions that specifically strain grounded language knowledge and self-other distinction (see Figure 1B). However, we assume that research progress can be accelerated by observing the problem from a perspective of the artificial self (Hafner et al., 2020; Nguyen et al., 2021) rather than disregarding the emerging properties as a side effect. The recent methods introduced in 3 provide important techniques that implement the required ingredients and are helpful in improving embodied dialogs and HRI applications. Still, we see a lack of methods that combine all of them jointly into one learning architecture.

Current RL methods without language representations can be extended with it (section 3.4.2), as they already include the skill learning phase (section 3.4.1). This is an important feature of RL because skill learning is a necessary prerequisite for language grounding. However, since language grounding is not a necessary prerequisite for skill learning, we conclude that RL-driven physical skill learning is more foundational for embodied dialog agents than disembodied language processing models like GPT-3 (Brown et al., 2020).

In the remainder of this section, we summarize the computational components that are important to develop embodied dialog agents based on self-representations. In addition, we provide references to successful implementations of these components. We subdivide these components into those that are related to predictive processes and those that are related to self-other distinction.

### 4.1. Predictive Processes and Crossmodal Self-Representations

Many methods compute prediction errors with inverse- and forward models that implement action-effect associations [e.g., Schillaci et al., 2016; Röder et al., 2020 and also neuroscience-related work like (Kaplan, 2007; Kidd and Hayden, 2015)]. At training time, these errors yield a signal for intrinsic motivation, helping to shape and update the body schema and sense of agency (see section 2.1). We see plenty of methods that implement this as curiosity-driven learning (Pathak et al., 2017; Nguyen et al., 2020; Akakzia et al., 2021; Hill et al., 2021). Other researchers model the prediction error not only with the sensory state but based on language. For example, Hermann et al. (2017) and Hill et al. (2021) consider word predictions given the egocentric view of an agent in a 3D environment. Hermann et al. (2017) predict a word at each time step, while a meaningful word of the current instruction serves as a target, e.g., the object “*apple”* given the instruction “*Pick up the red apple.”* This auxiliary task helps to shape the agent's representation in learning instruction to word mappings. Hill et al. (2021) compute a surprise score for both vision and language. An episodic memory with a specific language to vision key-mapping, inspired by dual-coding theory (Paivio, 1969), is queried to calculate a language- and vision-based distance as an intrinsic reward. Although this seems to be a promising approach, it is essential to consider some sort of weighting (Hill et al., 2021).

The authors empirically show that the less frequently encountered language is more important than the more frequently changing visual information. However, they are not using an appropriate body representation (Pathak et al., 2017; Nguyen et al., 2020) for the vision encoding to omit the *Noisy-TV Problem* (Burda et al., 2019), which might be the reason for the superior performance when using intrinsic rewards based on language only. Dean et al. (2020) implement an audio-visual association model to employ curiosity-driven exploration by exploiting the associations of two modalities, namely audio and vision.

The approaches above combine crossmodal integration in curiosity-driven and goal-directed learning procedures crucial for intelligent explorative behaviors (Georgie et al., 2019). When evaluating a trained agent, the internal models disclose metrics of surprise where the agent encounters dynamics that are novel or uncertainties with understanding instructions.

**Figure 5:**
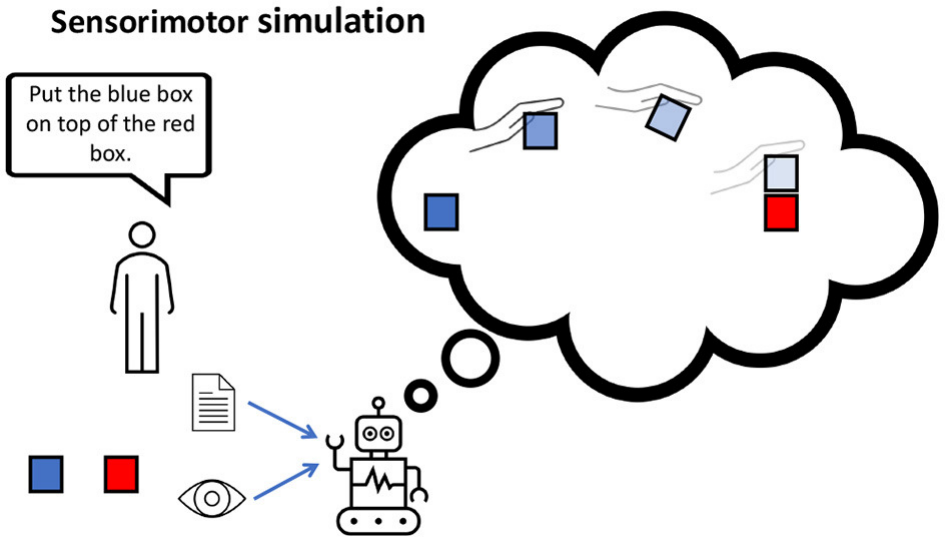
Internal models are capable of mentally simulating possible action trajectories given the visual observation and instruction of stacking the blocks. The longer the simulation horizon, the more uncertain the agent is about its predicted action-effects (illustrated with increasing color transparency).

Other important computational components for embodied dialog agents include hierarchical abstraction (Eppe et al., 2020) and automatically generated subtasks (Jiang et al., 2019) or latent plans (Lynch and Sermanet, 2021) to abstract away from low-level motor execution, toward higher-level conceptual representations. Abstractions are important because they limit the horizon of predictive processes. For example, in Figure 5, we illustrate sensorimotor simulation, using the internal model to unroll a latent (abstract) plan consisting of four steps only. If the same plan was represented in more fine-grained lower-level motor actions, this would lead to many more consecutive simulation steps, resulting in a higher cumulative prediction errors. Also, since predictions become less accurate the farther they are in the future, regenerating plans and subtasks happen more frequently. For example, Lynch and Sermanet (2021) use a hierarchy with a high-level module (plan encoder) to generate a latent plan at the frequency of 1 Hz, while a low-level action module (plan decoder) is executing motor controls at a frequency of 30 Hz. Similarly, the implementation of (Jiang et al., 2019) employs a 2-layer hierarchy that effectively leverages the compositionality of language to solve a task by solving subtasks.

Finally, having access to the agents internal hierarchical predictive state also allows observing metrics such as surprise and uncertainty (e.g., by measuring the prediction error) that expose how strong the sense of body ownership and agency is (Georgie et al., 2019; Hafner et al., 2020).

### 4.2. Self-Other Distinction

The scenario of Figure 1B requires the agent to understand the meaning of self-related words like *you* and other related words like *me*. Georgie et al. (2019) propose that distinguishing self-generated from externally produced sensational actions-effects are inevitable for an artificial self. By dividing the training procedure into two phases (section 3.4), agents learn the required body representations as describe by Georgie et al. (2019), Nguyen et al. (2021), and Hafner et al. (2020). The authors consider motor babbling as an active self-exploration process, starting with self-touch in prenatal development up to toddlerhood. Considering the progression from this early stage, the evolved body ownership and sense of agency define the minimal self (Georgie et al., 2019). We suppose that this stage is covered by our first phase (Figures 4A,B), employing motor babbling to train the internal models and motor skills from scratch.

The language-grounding phase (section 3.4.2) exploits the learned behaviors and body representations. This can be performed with a social partner or hindsight instructions to annotate behaviors. With the sense of body ownership developed during the skill learning phase, through minimal prediction error or free energy of inverse- and forward models, the agent can align its motor skills with grounded language. Social-psychological scientists like Mead et al. (2000) postulate the emergence of a self requires a social process based on the social theory of *symbolic interactionism*. However, there are limitations and different perspectives (Aksan et al., 2009) toward social RL (Jaques et al., 2019) and grounded language in a social context (Bisk et al., 2020). We consider these as future work and out of the scope of this article. Nevertheless, according to symbolic interactionism, self-awareness is a kind of reflection and inference of the behavioral observation of others. In other words, the self develops as a generalization of others, putting perception and expectations into the perspective of the social partners or group (Mead et al., 2000). This process allows sharing the same common understanding and thus the same language.

Despite the potential importance of social interaction, our review in 3 reveals that only Chevalier-Boisvert et al. (2019) contain some sort of interactive partner or teacher that provides linguistic and demonstrative feedback. The authors use a 2D environment and employ a synthetic simplified language (section 3.3). We suggest two possibilities to enhance the integration of a social partner to train a self-aware agent for communication.

The first possibility follows the approach of Chevalier-Boisvert et al. (2019), where the language grounding phase integrates a social partner, caretaker, or teacher. This agent supplies language annotations in hindsight (Akakzia et al., 2021) and, in addition, serves as an embodied entity that provides perceptible demonstrations in combination with language. The second possibility to develop a self for embodied dialog agents is to introduce a third alignment phase (see section 3.4), similarly to the developmental process of section 2.3.3, that involves external crossmodal sensory inputs of a social partner and considers fine-tuning the present motor-linguistic skills of the previous phases (sections 3.4.1 and 3.4.2).

In both cases, the language must explicitly refer to the individuals. Sentences like “You put red on top of the blue” or “I put red on top of blue” are possible examples that allow observing self- and externally generated stimuli in the context of language (McClelland et al., 2020).

## 5. Conclusion

This review contributes to the development of artificial agents for embodied crossmodal dialog. Our main hypothesis is that an explicit self representation is a critical component to enable embodied language understanding, going beyond disembodied language processing as proposed in recent machine learning articles. Reinforcement learning seems particularly suitable, as it allows by definition to discover the environment in a self-explorative manner, similar to an infant shaping its body schema within a self-conducted reinforcement process. Like Lynch and Sermanet (2021) and Akakzia et al. (2021), we suggest splitting the training of an agent into two phases, namely skill learning and language grounding (section 3.4). These two methods are the only ones regarding an embodied robot in a 3D environment and integrate most of the plausible concepts (see 2 and Figure 3) with state-of-the-art performance for complex instruction following. After the skill learning phase, language is grounded in sensorimotor- and body representations, hence in essential parts of the artificial self (Hafner et al., 2020).

As our main result and contribution, we propose and summarize computational components to implement and model an artificial embodied dialog agent in 4. Here, we highlight self-related components and expand the decoupled two-phased learning to a setting with an embodied social partner.

This approach is underpinned in social-psychological science (Mead et al., 2000) and by recent findings in neurorobotics (Hafner et al., 2020; Nguyen et al., 2021) which emphasize the significance of learning socially with other agents. These benefits arise because self-awareness and natural communication are learned by distinguishing self-generated from external stimuli and being part of social interaction. We believe that explicit self-representations in artificial agents improve robustness, performance, and trust for conversational settings because the emergence of a self is a consequence of low-level interaction with its body and environment (Schillaci et al., 2016; Hafner et al., 2020) and high-level verbal/non-verbal social interactions (Mead et al., 2000).

In this article, we focus primarily on mechanistic cognitive models, but we are also aware of the valuable neuroscientific research that examines the use of the RL framework (Botvinick and Weinstein, 2014), grounded language (Friederici and Singer, 2015; Garagnani and Pulvermüller, 2016), and curiosity (Kaplan, 2007; Kidd and Hayden, 2015). Considering the integration these neuroscientific theories would add a valuable additional dimension to our future research.

A simulation of the self with artificial agents is another beneficial future research direction. For example, we can potentially gain more insights from attention-based mechanisms (Chaplot et al., 2018; Hill et al., 2019), enabling us to visualize the agent's internal state as a kind of gaze following and eye tracking [see Hill et al. (2019), how they visualize the attention weights of different neural network layers when processing language and vision]. Such research paves the ground for measuring and defining neurologically inspired low-level metrics of an artificial agent's self in the future.

## Author Contributions

FR and ME authored and conceptualized the major parts of this article. OÖ mainly authored and contributed to section 3, revised the manuscript, and was involved in discussions with FR and ME. PN provided feedback for FR to conceptualize the initial outline. SW contributed through active feedback and revisions. All authors contributed to the article and approved the submitted version.

## Conflict of Interest

The authors declare that the research was conducted in the absence of any commercial or financial relationships that could be construed as a potential conflict of interest.

## Publisher's Note

All claims expressed in this article are solely those of the authors and do not necessarily represent those of their affiliated organizations, or those of the publisher, the editors and the reviewers. Any product that may be evaluated in this article, or claim that may be made by its manufacturer, is not guaranteed or endorsed by the publisher.

## Footnotes

^1^This idea was recently used to learn robust and diverse behaviors in goal-directed RL (Akakzia et al., 2021; Lynch and Sermanet, 2021).
